# Psychosocial Recovery Coaching and the National Disability Insurance Scheme: Outcomes and their Alignment with the CHIME-D Recovery Framework

**DOI:** 10.1007/s10597-025-01477-6

**Published:** 2025-06-24

**Authors:** Aurora Elmes, Robert Campain, Chris Brown, Erin Wilson

**Affiliations:** The Centre for Social Impact, School of Business, Law and Entrepreneurship, Swinburne University of Technology, Mail H25, Cnr John and Wakefield Streets, PO Box 218, Hawthorn, Vic 3122 Australia

**Keywords:** CHIME-D recovery framework, National Disability Insurance Scheme, Outcomes, Psychosocial recovery coaching

## Abstract

Australia’s National Disability Insurance Scheme (NDIS) provides individualised funding to eligible people with disability to purchase required services and supports. However, people with psychosocial disability have experienced challenges in accessing these supports. In response, the NDIS introduced psychosocial recovery coaching as a ‘recovery oriented’ support for people with psychosocial disability. This study, based on research undertaken with an Australian organisation providing psychosocial recovery coaching, aims to identify outcomes of this support and how these relate to the CHIME-D recovery framework and broader evidence on peer-delivered mental health support. The study shows that overall, participant experiences and outcomes of psychosocial recovery coaching align strongly with the CHIME-D recovery framework, and with the NDIS stipulated goals and responsibilities of psychosocial recovery coaching service delivery. This paper contributes new knowledge on the outcomes of psychosocial recovery coaching and suggests that future iterations of NDIS psychosocial supports should align with personal recovery outcomes—including those identified by people with psychosocial disability.

## Introduction

The NDIS provides individualised funding packages to eligible people with disability to purchase required services and supports (Carey et al., 2017, 2018; Green & Mears, 2014; Wilson et al., 2022). People with psychosocial disability (disability arising from a mental health condition) have experienced challenges in accessing NDIS supports – such as barriers to utilising NDIS funds, difficulty navigating services and inflexibility around funding use (Devine et al., 2022; Hamilton et al., 2020; Kendrick et al., 2017). In response, the NDIS has taken several actions to meet the needs of people with psychosocial disability including introducing a psychosocial disability recovery-oriented framework; recruitment of planners with specialised psychosocial disability knowledge; and implementing psychosocial recovery coaching. Psychosocial recovery coaching was introduced under the NDIS in July 2020 as a ‘Recovery Oriented’ support for people with psychosocial disability, to “take more control of their lives and better manage complex challenges of day to day living” (NDIA, 2020, p 4). As of June 2023, there were 62,000 participants (10% of all NDIS participants) with a primary psychosocial disability (Commonwealth of Australia, 2023).

With the release of the 2023 NDIS Review Final Report, several changes to psychosocial supports, including the current form of psychosocial recovery coaching, have been recommended (Commonwealth of Australia, 2023). Given these potential changes, we explore how evidence on psychosocial recovery coaching outcomes can inform future iterations of NDIS psychosocial disability services. The paper evaluates psychosocial recovery coaching within the broader context of mental health peer-delivered supports, drawing on empirical research with psychosocial recovery coaching clients to identify the outcomes of psychosocial recovery coaching and how these relate to the CHIME-D recovery framework (van Weeghel et al., 2019), and evidence on other types of peer-delivered mental health support. Finally, we explore what learnings may inform future NDIS psychosocial disability support.

## Psychosocial Recovery Coaching – Context and Features

The development of psychosocial recovery coaching was informed by scholarly work and consultation across the disability sector on how the NDIS could provide more responsive services to people with psychosocial disability (e.g. Roberts & Fear, 2016), and address some of the challenges including barriers to utilising NDIS funds, difficulty navigating services, and lack of alignment with personal recovery frameworks (Devine et al., 2022; Hamilton et al., 2020; Kendrick et al., 2017). In their work on the NDIS and recovery oriented psychosocial disability support, Brophy et al. (2021) summarised the elements of ‘good’ recovery-oriented practice noting it should be social and relational, person-centred, human rights-focused, and aligned with recovery frameworks. In addition, good recovery-oriented practice should support hope, empowerment, navigation of services, and a personally meaningful life (Brophy et al., 2021). This aligns with the *NDIS Psychosocial Disability Recovery Oriented Framework* which among its principles highlights the valuing of lived experience, and the supporting of both personal recovery and informed decision making (NDIA, 2021).

Although the role of a psychosocial recovery coach may shift following recommendations of the recent NDIS Review, the directive of supporting people with psychosocial disability in their personal recovery remains (Commonwealth of Australia, 2023). This emphasis on *personal* recovery is important as it focuses on people living a meaningful life in the context of illness, as opposed to *clinical* recovery which focuses on the reduction or absence of mental health symptoms (NDIS, n.d., p.2).

Psychosocial recovery coaches offer capacity-building supports with a focus on coaching, developing and implementing recovery plans and collaborating with other services rather than providing core supports for activities of daily living, or community, social and recreational activities. In line with a personal recovery orientation, psychosocial recovery coaching responds to individual requirements and can be varied and tailored as needed over time, though in general it is “intended that recovery coaches will provide support to people with psychosocial disability to increase their independence, social participation and economic participation” (NDIA, 2020, p.4). The NDIS guidelines state that the psychosocial recovery coaching role includes building a recovery enabling relationship, supporting a sense of hope, skills and capacity, and assisting the person to engage with the NDIS and other services. This may include:

- capacity building supports to build skills, independence, and support decision making
- social skills development to help build relationships with family, friends and be part of the community
- building life skills including confidence, resilience, and taking care of health and wellbeing
- social and recreation support to join social activities, sporting clubs or community groups
- help to find somewhere to live and to manage rental or home ownership responsibilities
- support from allied health professionals
- help to plan and coordinate NDIS supports with mental health treatment and services

Skills required of a psychosocial recovery coach include knowledge of community and mainstream services. This enables them to connect and support their clients with health and mental health services outside the NDIS. These services should be integrated and align with a client’s recovery plan (NDIS, 2024).

In addition, psychosocial recovery coach competencies include being able to draw on lived (and/or learned) experience and knowledge to support and enable people in their recovery journey (NDIA, 2020, p.12), differentiating them “from support coordinators in that they bring knowledge and skills in psychosocial recovery, mental health and service navigation within the mental health system” (NDIA, 2020, p.10).

## Existing Evidence on Recovery Coaching

Given the relatively recent introduction of psychosocial recovery coaching and very limited scholarly literature on this service type, we draw on the broader evidence on recovery coaching, which largely refers to peer recovery support services for people with substance use disorder—though people may have another co-occurring mental health condition (Eddie et al., 2019)—and mental health recovery literature, which also contributes to recovery coaching and the use of peers to assist in recovery (Corrigan, 2024; Tari-Keresztes et al., 2022). While definitions of a recovery coach vary (Felton et al., 2023), lived experience is identified as an essential component by some scholars, building trust and rapport and instilling hope (Jack et al., 2018; Magidson et al., 2021; Simske et al., 2019 cited in Amat & Johari, 2024) while engaging in empathetic support to help people achieve their goals (Corrigan, 2024). Commonly described recovery coaching activities include providing “informational, emotional, social and practical support services” (Eddie et al., 2019, p.2; Byrne et al., 2024).

A recent review of peer recovery coaching for people with substance use disorder found that navigation of services was often the initial step in assisting people to connect with appropriate supports (Kang & Kang, 2022). There is evidence this kind of ‘navigator’ role is valued as part of providing coordinated care services and connecting to community resources (Davidson, 2021; Eddie et al., 2019). Other important features of peer recovery coaching include educating and supporting people to build ways of coping effectively with stress, developing strategies for dealing with difficulties (Kang & Kang, 2022), and providing “emotional support, counseling and case management services” (Kang & Kang, 2022, p.259). Finally, the substance use disorder literature suggests that peer recovery coaches seek to support people’s recovery both in terms of recovery *from* disordered substance use, and recovery *of* personally meaningful activities and life goals (Kang & Kang, 2022), while a systematic review of a range of studies shows tentative support for outcomes including reduced substance use and relapse rates, improved relationships with social supports and service providers, and increased treatment retention and satisfaction (Eddie et al., 2019).

Winsper et al’s. (2020) systematic review into mental health recovery-oriented interventions identified these interventions as contributing to mental health recovery with goal setting and empowerment as essential elements, while peer support is unique in providing role models of individual recovery. Peer support can facilitate mental health recovery through shared experience where voices are heard and valued without judgement (Tari-Keresztes et al., 2022). However, despite these encouraging findings, overall, the evidence on recovery coaching in both substance use disorder and mental health recovery is mixed, and drawing conclusions is challenging given interventions operate within highly complex systems which can moderate effectiveness. There is also variability within the literature on the nature of recovery supports and definitions – including whether recovery coaching is delivered by peers, as well as who is receiving services and how outcomes are evaluated (Eddie et al., 2019; Winsper et al., 2020). 

Amat and Johari’s (2024) examination of the evidence concludes that the existing literature provides strong evidence for the short-term benefits of recovery coaching with addiction recovery, though with limited evidence as to its effectiveness over prolonged periods.

Following the introduction of psychosocial recovery coaching as a service under the National Disability Insurance Scheme, the evidence to date is limited. Existing evidence focuses on the NDIS and the relationship with the mental health sector and the barriers to accessing the scheme rather than the practice of recovery coaching (Devine et al., 2021; Hancock et al., 2022; Mellifont et al., 2023; Stewart et al., 2020). One qualitative study (Roberts et al., 2024) based on interviews with people with psychosocial disability accessing the NDIS, identified one key outcome in highlighting the value of support workers in providing social connection and engaging with the social world—an essential element in the recovery process.

Overall, however, there is a lack of psychosocial recovery coaching evidence, with a review of published literature on the NDIS and mental illness/psychosocial disability identifying there is ‘limited peer-reviewed literature reporting on experiences and outcomes of the Scheme for people living with psychosocial disability’ (Roberts et al., 2024, p. 1162). The authors note the need for further research on this topic for future development of the NDIS. Hence this research aims to address this limitation, with the focus on recovery coaching as introduced through the NDIS as an identified need for people with psychosocial disability.

## Methods

The study sought to answer the following questions:

1. What changes or outcomes does psychosocial recovery coaching support participants with?
2. To what extent are participants achieving good outcomes through psychosocial recovery coaching?
3. What are the barriers to achieving desired changes or outcomes through psychosocial recovery coaching?

This involved interviews with ten recovery coaching clients, and an outcomes-focused survey of 49 clients from multiple One Good* Day service locations across Australia. One Good* Day (OG*D) is an organisation providing psychosocial recovery coaching throughout Australia to people with a psychosocial disability whose NDIS plan enables access to this support. The organisation employs psychosocial recovery coaches with lived experience related to mental health, alongside learned expertise obtained through mental health qualifications and/or working histories in the sector. Approximately 75% of OG*D team members identify their own lived experience with mental health and recovery. Further OG*D staff characteristics (as of 2022) included:

- 83% of staff were Recovery Coaches
- The largest proportion of staff (49%) were based in Victoria
- 77% of staff were female
- 66% of staff were aged between 26–45 (OG*D, 2023).

Recruitment for this study was via OG*D who distributed the relevant material to their clients on behalf of the researchers, with respondents contacting researchers directly (interviews) or completing the survey via a survey link. As psychosocial recovery coaching is a new type of service with limited outcomes data, we conducted interviews with psychosocial recovery coaching clients early in the project to explore and learn from participants’ own experiences and descriptions of psychosocial recovery coaching and its influence on personal recovery processes. This approach aligns with recommendations for research on recovery focused services to include qualitative data on participants’ perspectives (Corrigan et al., 2022).

The CSI Swinburne research team conducted the client interviews between October and November 2022 via phone-call or online meeting. Interview questions asked about: clients’ experience of recovery coaching; what difference recovery coaching made for clients and what outcomes they were supported with; how recovery coaching supported clients’ recovery; what had been particularly helpful or unhelpful; and what questions should be asked to understand whether recovery coaching is working for people.

Drawing on the themes raised in these client interviews, the research team collaborated with the two founders of OG*D to determine the outcomes and questions used in the client outcomes survey. These outcomes were based on the Community Services Outcomes Tree (CSOT) – an analytic framework to help community sector organisations measure the effect they are having on individuals’ lives. It comprises 12 life domains which contain a number of related outcomes (over 100 in total). The framework encourages a ‘whole of life’ approach and recognises the way in which life domains interrelate. The framework has been used across a range of community services to measure impact (Wilson et al., 2021; Wilson et al., 2024).

For the brevity of the survey, decisions were made as to the most appropriate outcomes – eleven in total from 6 of the twelve CSOT domains (Table 1). Common themes identified across the interviews with psychosocial recovery coaching clients informed the outcome items included in the survey—for example “Social support” and “Building confidence in navigating the NDIS”. Based on input from client interviews, the survey also included questions about whether the psychosocial recovery coach was a good match and built a positive relationship with the client, and whether clients got what they had hoped for from psychosocial recovery coaching. The survey also asked about: outcomes (changes in areas of life); contribution of the OG*D service to outcomes; barriers to outcomes; service improvements; basic demographics and length of service provision. The list of barriers to outcomes was also drawn from the CSOT tool and reviewed based on the interview analysis.

**Table 1:** CSOT: Survey outcomes and associated domains

Outcome	Domain
1. Meaning and purpose	Daily life
2. Choice and control in daily life 3. Self-reliance and resilience 4. Building confidence in navigating the NDIS (Choosing supports and services)	Choice and empowerment
5. Social emotional health 6. Mental health	Health
7. Social support 8. Feeling valued and respected	Social inclusion
9. Understanding and using the NDIS funds I have (Access to/use of services) 10. Access to/use of other non-NDIS services (Access to/use of services)	Services and government benefits
11. Employment (General)	Employment

Some outcomes (e.g. outcome 4, 9, and 10 in Table 1) have used wording customised to psychosocial recovery coaching within the NDIS, with their alignment to established CSOT outcomes outlined in brackets (Table 1)

In addition to the 11 outcomes, an “Other” outcome option was included where survey participants could self-describe any other outcomes they experienced.

Survey respondents reported how outcomes had changed since receiving psychosocial recovery coaching based on a scale ranging from:

Got a lot worse; Got a bit worse; Not changed; Got a bit better; Got a lot better; Not relevant.

The proportion of respondents reporting positive outcomes (defined as ‘got a bit better’ or ‘got a lot better’), negative outcomes (defined as ‘got a bit worse’ or ‘got a lot worse’) or ‘no change’ is presented for each questionnaire item in the Findings section.

Ethics approval was obtained from the Swinburne University Human Research Ethics Committee on 10 October 2022 (No. 20226732–10817). In line with ethics requirements, informed consent was obtained from all individual participants included in the study, including consent to publish the results in academic publications.

### Data Analysis

In keeping with consumer advocacy for evaluation of mental health supports to focus on personal recovery (Brophy et al., 2021), this paper analyses the interviews and outcomes data using the CHIME-D recovery framework (van Weeghel et al., 2019). The CHIME framework of personal recovery—which was designed to capture key elements of personal recovery including Connectedness, Hope and optimism, Identity, Meaning in life and Empowerment (Leamy et al., 2011)—is widely supported within the literature on personal recovery, with a review by van Weeghel et al. (2019) recommending the inclusion of a further aspect: “Difficulties and trauma” that emphasizes the challenges people have or will encounter in life and how people make choices to navigate and cope with these. While our study survey broke outcomes into discreet items for clear participant understanding and consideration, drawing on the CHIME-D in data analysis allows us to consider this data in the context of an established personal recovery framework, which unearths the key themes of support emphasised in psychosocial recovery coaching and the ways these contribute to processes of personal recovery.

Interviews were analysed abductively by theme (Dubois & Gadde, 2002) involving three researchers separately conducting analysis of interview data using NVivo and manual coding, before reviewing each other’s analysis to see where themes aligned or differed. Themes were discussed and refined by consensus among the research team. This thematic analysis was aligned with both the CSOT and CHIME-D and identified the relation between the two frameworks. Thematic analysis is an inherently subjective and interpretive practice, emphasising researcher reflexivity. Coding can never be accurate in a positivist scientific sense. Meaning is not ‘fixed’ within the data and is always reliant on the reader/researcher interpretation and construction (Braun & Clarke, 2023). In this instance, coding reliability is determined by the use of analytical frameworks with themes aligning to both the CSOT and CHIME-D with the use of multiple coders to ‘sense’ check ideas and interpretations of the data. In this way, it is argued that the results provide a meaningful and insightful understanding of the topic and people’s experience of it, while also recognising that ‘qualitative analysis as a whole does not contend to provide a single or ‘correct’ answer’ (Braun & Clarke, 2013).

Table 2 shows the outcomes used within the client survey, and their alignment to corresponding aspects of the CHIME-D recovery framework. While there is not a one-to-one direct correlation between discreet outcomes and broader CHIME-D concepts, it is apparent that both the survey and CHIME-D capture similar life elements. Where survey outcomes align with multiple CHIME-D categories, we have grouped them with the most similar elements of CHIME-D.

**Table 2:** Survey outcomes corresponding to the CHIME-D recovery framework

Survey outcomes	CHIME-D recovery framework
Meaning and purpose	Meaning in life
Choice and control in daily life (e.g. feeling more in control, doing things I want to do, choice in daily activities. Learning things, solving problems and making decisions for myself)	Empowerment
Self-reliance and resilience (e.g. feel motivated to do things, cope with demands and unexpected events)	Hope and optimism; Coping with difficulties and trauma
Building confidence in navigating the NDIS (e.g. having the information and advice needed to support productive engagement with the NDIA and better navigate the NDIS. For example, appropriately preparing for your plan review)	Empowerment
Social emotional health (e.g. feeling more confident, greater self-esteem and self-belief, improved communication)	Identity
Mental health (e.g. feeling more relaxed, less anxious and depressed, feeling less ‘stuck’)	Hope and optimism; Coping with difficulties and trauma
Social support (e.g. less isolated – more socially connected and not alone. Have someone to trust, talk to and who listens)	Connectedness, Hope and optimism
Feeling valued and respected (e.g. being listened to, valued and respected by those who support me)	Connectedness
Understanding and using the NDIS funds I have (e.g. help with tracking or managing how NDIS funding is used, support with finding and getting the NDIS-funded services needed)	Empowerment, Hope and optimism
Access to/use of other non-NDIS services (e.g. GP, dentist, housing services, etc.)	Empowerment
Employment (e.g. career planning; job skills; work experience; positive work attitudes and behaviours; gain employment; maintain employment)	Identity, Meaning in life

### Reliability and Trustworthiness

We sought to ensure reliability and trustworthiness across all aspects of the research to ensure a rigorous process that provides validity to the findings and to minimise researcher bias in interpretation and study construction. Survey design was undertaken in consultation with service clients who provided feedback on a survey draft with amendments made in accordance with recommendations. Methodological triangulation was used with both survey and interview questions designed for respondents to share both what is working and what isn’t working with regard to psychosocial recovery coaching. The researchers placed emphasis on the confidentiality and anonymity of individual responses to promote more reliable discussion on this sensitive topic. Interview questions were open-ended and consistent across all interviewees, and interviewers sought to avoid offering opinions or asking the questions in a manner that encouraged respondents to answer in a certain way. Interviewees were provided with the opportunity to review and correct the interview transcript. The research team was comprised of three interdisciplinary members who conducted their own separate analysis, providing inter-rater reliability as a measure of consistency and agreement among the researchers in their assessments. Reflexive conversations enabled the opportunity to work through any disagreements and to further refine themes and the overall analysis.

## Findings: Demographics

### Survey Participants

Over half of survey respondents were from Victoria (55%). Half (50%) of participants were female, 39% were male, 7% used another gender descriptor and 4% were non-binary. Most participants (80%) were aged 36 or older; with the largest proportion of participants aged between 36–45 (30%) and 46–55 (27%). Approximately 20% were aged 35 or less. One survey respondent identified as being of Aboriginal or Torres Strait Islander origin.

### Interviewees

Of the ten clients interviewed, the majority (90%) were based in the State of Victoria and 10% in Queensland. Seventy percent (70%) were female, and 30% were male. Fifty percent (50%) identified their cultural background as Australian, while another 40% identified with at least one other cultural background (the remaining 10% found this question hard to answer). Most participants (90%) were aged between 26–55 with the highest proportion (40%) aged between 26–35. One person (10%) was aged over 55.

Overall, interviewees were broadly representative of One Good* Day client demographics in terms of location, gender and age though the data is skewed towards a Victorian cohort. Caution needs to be exercised when considering how representative findings are across the Australian context.

## Findings

### Positive Outcomes of Recovery Coaching

**Fig. 1:**
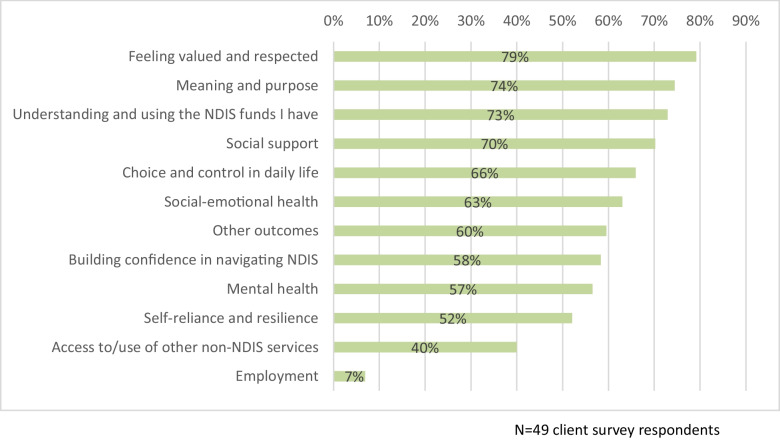
Survey outcomes: Respondents who reported positive outcomes since receiving recovery coaching from OG*D

Overall, most participants reported positive outcomes. Of the 12 outcome areas included in the survey of recovery coaching clients, 10 received positive change ratings from more than half of the participants (Fig. 1). Less than half of the participants reported positive outcomes for ‘Access to/use of other non-NDIS services’ (40%) and ‘Employment’ (7%).

We report on psychosocial recovery coaching outcomes from both the survey and the client interviews using the structure of the van Weeghel et al. (2019) CHIME-D recovery framework – Connectedness, Hope and optimism, Identity, Meaning in life, Empowerment, and Coping with difficulties and trauma. Personal recovery is often described as a process (van Weeghel et al., 2019), though process and outcomes can overlap and be somewhat indistinct. For example, connectedness was described through both the process of establishing a consistent and positive relationship with the recovery coach, leading to participants feeling valued and respected, and contributing to outcomes of improved social support and community participation. This blurring between factors that contribute to recovery (e.g. social support), and aspects of recovery (connectedness) has been previously recognized in the literature (Roberts et al., 2024; van Weeghel et al., 2019).

Quotes used throughout the presentation of results are used to illustrate both the survey findings and the thematic analysis based on CHIME-D categories.

#### Connectedness

Connectedness is described in the personal recovery literature as (re)building relationships, having support from peers, and being part of society (Leamy et al., 2011, in van Weeghel et al., 2019). Connectedness was often experienced through a trusting and supportive psychosocial recovery coaching relationship. **Feeling valued and respected** was the most frequently reported positive outcome among clients, with 79% reporting this as a positive outcome. The majority (70%) also reported improved **Social support**. Participant comments illustrated that (particularly for socially isolated participants) the consistent connection and relationship with their psychosocial recovery coach was itself a valued form of social support. Knowing their coach could be contacted as needed is helpful for clients, rather than only having structured appointments without flexibility.

*I feel like I’ve got someone I can talk to and rely on always… If you don’t have someone you can talk to about your problems – [you] can’t get anywhere in life. – Client interview 6*

*I feel more supported and less alone. I know that my RC [Recovery Coach] will assist me in things I find challenging (e.g. dealing with Centrelink, finding supports) and will be there when it feels overwhelming… – Client survey respondent*

For others, being supported to access community groups, supports or activities expanded their social connectedness.

*I have got a lot more support and am able to access some recreational activities that really help my wellbeing and I feel less alone. – Client survey respondent*

*Because a lot of my issues are with anxiety and depression… to be able to connect with the community and have someone facilitate that is really handy. – Client interview 8*

The positive outcomes identified by clients suggest psychosocial recovery coaching is contributing to clients’ sense of connectedness and support, and meeting several key aims of psychosocial recovery coaching as set out by the NDIA (2020).

#### Hope and Optimism

Within the context of personal recovery, hope and optimism might include a sense of motivation, developing goals and thinking positively about one’s capacity to progress towards these (Honey et al., 2023; Leamy et al., 2011, in van Weeghel et al., 2019). Hope and optimism were often described in relation to the consistent psychosocial recovery coaching relationship, being able to reach out for help as needed, engaging in encouraging conversations and knowing support is there.

*[My recovery coach] gives me the hope and the reassurance that, you know, if today isn’t a great day, tomorrow can be better… yeah, it gives me a lot more hope than I may have in myself which is good. – Client interview 7*

For some, being supported to access other services such as medical appointments, cleaning and gardening enabled greater optimism in ability to cope with the demands of life.

*“Things that seemed impossible are now looking more achievable”. - Client survey respondent*

*Just having that progress. Even though it’s little steps most of the time, I just sit back now and think… “Okay, things aren’t perfect here by any means but—you know what—I have a clean house now, I have a gardener that comes so my lawn isn’t fence-high anymore”. Just all those little things—when your environment’s all chaotic and out of control you can’t even start to focus on other things because everything’s just craziness. To now have a clean house and to have a garden that I can go outside in—things like that is just massive.—Client interview 5*

#### Identity

Within a personal recovery framework, identity is described as having a positive sense of self (Leamy et al., 2011; van Weeghel et al., 2019). Identity was a less common theme throughout our findings, but clients did discuss some aspects of how psychosocial recovery coaching supported their goals relating to self-identity. Some aspects of identity (such as positive sense of self, self-esteem and confidence) also showed evidence of improvement (**Social and emotional health**: 63% of respondents). Six out of ten clients who were interviewed talked about psychosocial recovery coaching contributing to increased confidence or a more positive sense of self. The most common identity-related theme across the interviews and survey was increased ability to pursue interests and goals.

*My recovery coach has gradually moved me into more activities, which has increased my confidence with people. – Client survey respondent*

*[The recovery coaching has] sort of boosted my confidence a lot… Having the confidence – I mean, that's something that I never had because of low self-esteem. I think it's very, very important in your state of mind… because there's been times I haven't been able to go out the front door—but now I feel more confident more or less in doing that – Client interview 3*

#### Meaning in Life

Meaning in life relates to identifying and having opportunities to pursue activities or goals that are personally meaningful (Leamy et al., 2011; van Weeghel et al., 2019). Survey and interview findings indicated that with psychosocial recovery coaching support, clients increased their capacity to work towards goals and spend time on what’s important to them – an alignment with the NDIA-stated purpose of supporting people in developing goals for their life through recovery planning (NDIA, 2020). In total, 74% of survey respondents rated positive outcomes related to **Meaning and purpose**. This is consistent with interview findings where 6/10 clients identified outcomes around freedom to focus on life goals.

Participant comments described pursuing meaningful activities or progressing towards goals that provided a greater sense of purpose and direction in life.

*I think things are improving in the sense that I've got a little bit more of a life. My husband and I are hoping to find our own house and we're looking into getting a puppy. – Client interview 1*

*I feel so much more hopeful about my future and that I can have the sort of life that's important and meaningful to me. – Client survey respondent*

Conversations about goals reconnect clients with what is meaningful to them and what they want in life and the incremental steps towards achieving it, along with access to material support to help remove financial barriers to full participation.

*Goal setting…It's like. “OK, well, so I'm taking these tiny little steps even if I haven't done it”. It's more in my consciousness, so I'm closer to being able to…complete these goals. – Client interview 4*

*My coach has taught me what is possible to ask for to use my funding well. And we are working toward my long-term goals of writing and getting my driver’s license. I feel happy that things are finally changing for me. – Client survey respondent*

#### Empowerment

Empowerment is described in the recovery literature as building and focusing on strengths and capacity to respond to one’s own needs and aspirations (Leamy et al., 2011; van Weeghel et al., 2019). Themes of empowerment often related to psychosocial recovery coaches sharing knowledge, providing relevant support options aligned with clients’ needs and goals, and encouraging clients to express preferences and feedback. Survey respondents identified several improved outcomes in relation to the NDIS, including 73% of respondents experiencing improvement in **Understanding and using the NDIS funds I have**, and 58% experiencing improvement in **Building confidence in navigating the NDIS**.

*NDIS has changed my quality of life. The advice and guidance in navigating the NDIS from my recovery coach has been invaluable. – Client survey respondent*

*[The biggest change has been] help to navigate the NDIS. It's time consuming and challenging to do on your own. The recovery coach has enabled me to access supports in my local area at times that work for me. – Client survey respondent*

Similarly, interviewees also highlighted gaining access to needed services (8/10 clients); and decreased barriers to NDIS navigation (7/10 clients). This highlights the value of personal support with knowledge of the NDIS given the difficulties people often face in navigating the Scheme (Carey et al., 2018; Roberts et al., 2024; Wilson et al., 2022).

Both interview and survey data indicate that psychosocial recovery coaches support empowerment of people with psychosocial disability through enabling decision making by eliciting client priorities and preferences and providing relevant information to support decisions (7/10 clients interviewed). There was evidence that the NDIA stipulated responsibility of “providing coaching to increase recovery skills and personal capacity, including motivation, strengths, resilience and decision-making” (National Disability Insurance Agency, 2020) was contributing to positive outcomes.

About two thirds (66%) of survey respondents reported improvements in **Choice and control in daily life**. Affirming clients’ right to assert choice, and providing information that supports this, aligns with the NDIA psychosocial recovery coaching purpose of helping the person’s understanding of human rights and capacity-building for self-advocacy.

*I've been getting so much help in terms of the services that they've set up for me. And there's been times where my [recovery coach] would say, “Look, these are all the different ways, all the different things that could benefit”. That's been really helpful… – Client interview 1*

*Yeah, to just step in and say, “Yep, we’ll get it done”—without feeling like she’s controlling, if that makes sense? She’s done some research with what I said I need, and then she’s brought me back choices. So it’s not like she’s just gone, “this is the one that’s going to work for you”. She’s still given me choices. – Client interview 5*

#### Coping with Difficulties and Trauma

Coping with difficulties and trauma is identified as an important aspect of personal recovery (van Weeghel et al., 2019), and our study suggests psychosocial recovery coaching can contribute to participants’ capacity to cope. Just over half of survey and interview participants reported improvements in coping with difficulties through being able to seek support from their coach, or problem-solve how needs could be met. In total, 57% of survey respondents identified positive outcomes related to **Mental health.** In addition, survey findings showed that **Self-reliance and resilience** had improved for 52% of respondents. These findings were consistent with interview findings, where 6/10 clients discussed the positive impacts of psychosocial recovery coaching in supporting them to build strategies to manage mental health.

*I am provided with a caring, empathetic and understanding recovery coach who is genuine and I feel I can trust and talk to. She provides me with a listening ear and encouragement which I value and gives me greater confidence to be independent. I am living more harmoniously with others and have the ability to stand up for myself now. – Client survey respondent*

*My suicidal ideations have stopped, because I finally have the right supports in place. I cannot emphasize this enough, but having a recovery coach and caring supports has saved my life. – Client survey respondent*

However, not all participants experienced improvements in coping or mental health outcomes, as discussed further in the next section.

### Negative Outcomes or No Change

**Fig. 2:**
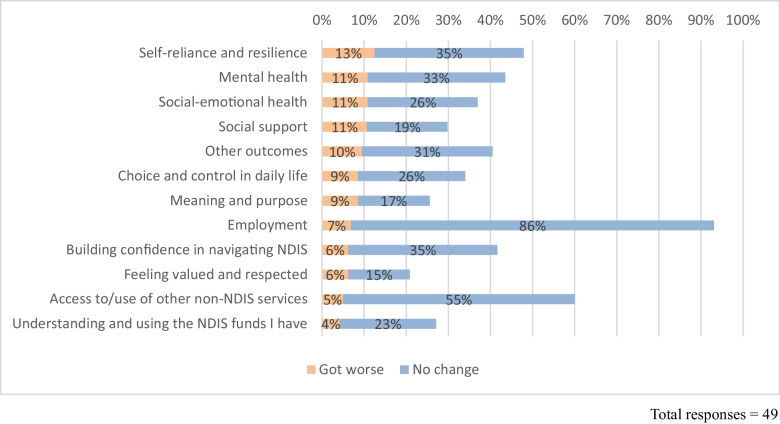
Respondents who reported negative outcomes or no change since receiving recovery coaching from OG*D

While our findings indicate that psychosocial recovery coaching yields largely positive outcomes aligned with the CHIME-D recovery framework (van Weeghel et al., 2019), expectations set out in the NDIA psychosocial recovery coaching guidelines, and with findings of previous literature on peer delivered support (Corrigan et al., 2022; King & Simmons, 2018; Simmons et al., 2023), there were some participants indicating less positive change than others. Across the outcome areas explored through the survey and client interviews, a proportion of people reported no change and some reported negative changes. Figure 2 shows the proportions of survey respondents reporting ‘no change’ or negative change for each outcome area in the survey. Some caution needs to be applied in interpreting ‘no change’ as it cannot be determined to what extent change was sought and whether ‘no change’ may indicate welcome stability. Further data is required to more comprehensively understand the significance.

The outcome with the highest proportion of survey respondents reporting ‘no change’ was **Employment** (86%). While this is a high proportion, gaining or changing employment may not have been a specified goal for all participants – further information is required to understand this result. The next outcome with the highest proportion of people reporting no change was **Access to/use of other non-NDIS services** (55% reported ‘no change’). Between a quarter to a third of survey respondents reported ‘no change’ for two outcomes related to NDIS utilisation—including: **Understanding and using the NDIS funds I have** (23%) and **Building confidence in navigating the NDIS** (35%). In line with recent research (Dickinson & Yates, 2023), comments from both the client survey and interviews indicate that NDIS complexity and inflexibility represent a structural barrier to empowerment that impacts participants’ capacity to understand and navigate the NDIS without support.

*I’m just like, I don’t even know this whole NDIS thing is for me. It’s just making me frustrated and if the recovery coaches are overwhelmed, and I’ve got all these supports and if it’s not working, can anybody help me? - Client interview 5*

Approximately a third of respondents indicated ‘no change’ for **Self-reliance and resilience** (35%) and **Mental health** (33%). In addition, a minority of respondents reported negative outcomes since receiving psychosocial recovery coaching. In some cases, participants linked their lack of progress or negative change to service-related issues such as staffing changes, while others mentioned health challenges or life circumstances.

The following section elaborates on the factors participants identified as barriers to change.

### Barriers to Achieving Desired Changes or Outcomes

**Fig. 3:**
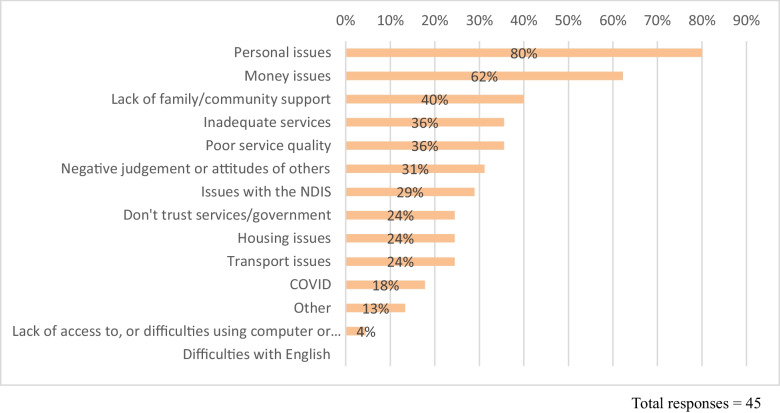
Barriers to positive outcomes as reported by respondents

While both clients and service providers are invested in achievement of desired outcomes, barriers of a structural, service-related or personal nature can impact on attaining these. The literature on personal recovery identifies common recovery barriers as social and economic exclusion, stigma, lack of supportive relationships, impacts of mental health symptoms, and negative service experiences (van Weeghel et al., 2019; Wilson et al., 2022). These factors contribute to the difficulties and trauma people experience, and despite the range of coping strategies people may employ, can impede people’s recovery. Survey respondents indicated from a list of potential barriers what they viewed as the biggest barriers to achieving positive outcomes (Fig. 3).

Survey respondents most frequently identified the biggest barriers as **Personal Issues** (80%) and **Money issues** (62%). **Lack of family/community support** was also reported as a barrier by 40% of survey respondents. While survey respondents did identify **Lack of family/community support (40%)** and **Negative judgement or attitudes of others (31%)** as barriers, neither of these was significantly identified in interviews.

#### Personal Issues, Including Impacts of Mental Health Conditions

Personal issues were identified as one of the biggest barriers for 80% of survey respondents, and through survey text comments nearly half (49%) of respondents reported mental health and lack of confidence as a major barrier. It’s important to emphasise that these ‘personal issues’ are compounded by structural barriers that affect people’s lives, such as inadequate support with mental health concerns (Roberts et al., 2024; Wilson et al., 2022). Survey respondents and clients who were interviewed described these kinds of barriers in a range of ways:

*Mental illness and lack of motivation and organisational skills due to mental illness. Not having supports in place or people around me that were understanding of my mental illness. – Client survey respondent*

*Although I am making positive changes, there have definitely been barriers along the way which my recovery coach has been supporting me to address. However, the biggest barrier for me is my history of trauma and the psychosocial disabilities that I live with. – Client survey respondent*

#### Low Income (Including Poverty/Lack of Employment)

Money issues (62%) were the next most frequently identified barrier to achieving outcomes. Some respondents specified that money was a barrier to achieving goals such as community participation and could also be a barrier to accessing the right level of services to adequately support wellbeing. Others reflected that lack of appropriate or inclusive employment opportunities was a barrier.

*For example one of my goals is to get out in the community, though I can not afford to do any activities in the community. – Client survey respondent*

*The biggest barrier is that we still have yet to find a job that I can do with my health limitations. – Client survey respondent*

#### Issues with Services or Supports

Participants identified that both inadequate services (36%) and poor service quality (36%) were barriers to successful outcomes. The barriers to outcomes reported by participants primarily encompass broader structural issues around access to adequate resources, support and services, and the ways in which these conditions interact with participants’ lives.

*When I find what I want, I can't have what I want. Limitations on services, supplies, resources; I don't know what's possible. – Client survey respondent*

*Stress of dealing with Centrelink [income support agency]. Finding it hard to find a support worker (have a good person now). Poor quality of supports I previously tried—and little assistance from previous RC's [recovery coaches]. Setbacks due to stresses of dealing with certain people. – Client survey respondent*

#### The NDIS – System Complexity and Inflexibility

Survey respondents highlighted complexity and inflexibility of the NDIS as an impediment to accessing and receiving appropriate supports—findings consistent with other related studies (Commonwealth of Australia, 2023; Roberts et al., 2024; Wilson et al., 2022).

*The NDIS. Holy hell I've never experienced a group that were so hell bent on making me more sick. – Client survey respondent*

*NDIS inflexibility, doesn't understand impact of all my health conditions, only some, doesn't understand how physical and mental health issues affect and reinforce each other. – Client survey respondent*

Respondents suggested that more autonomy in how funds can be used would enable participants to get the most benefit from their NDIS plan.

*The barrier is that NDIS does not give me autonomy to allocate my funds where they would be most helpful, despite me providing robust supporting letters, justification and recommendations from several highly skilled and qualified health professionals. – Client survey respondent*

## Discussion

This study responds to the paucity of research into psychosocial recovery coaching, by focusing on the outcomes of psychosocial recovery coaching, and the barriers that inhibit successful outcomes. In doing so, the research aims to inform implications for service design and for the NDIS, and to meet the requirements of people with psychosocial disability. The study provides initial evidence that psychosocial recovery coaching contributes positively to many of the aspects of personal recovery articulated in the CHIME-D framework (van Weeghel et al., 2019). Our findings suggest that, similar to other recovery-oriented peer-delivered services, psychosocial recovery coaching can contribute to recovery-related outcomes such as connectedness, hope, empowerment and coping with difficulties (King & Simmons, 2018; Lloyd-Evans et al., 2014; Pitt et al., 2013; Repper & Carter, 2011), illness management (Corrigan et al., 2022; King & Simmons, 2018; Simmons et al., 2023), and perceived health and service access (Corrigan et al., 2022).

The outcomes showing the most evidence of positive change in our study included connectedness, sense of meaning and purpose in life, empowerment in the use of NDIS services, and choice and control in daily life. This is particularly pertinent given that NDIS participants with psychosocial disability experience lower community participation and employment than other NDIS participants (Commonwealth of Australia, 2023), and NDIS services have been challenging to access and use for this cohort of participants (Devine et al., 2022). Connectedness was experienced both through feeling valued and respected by the psychosocial recovery coach and through improvements in social support – outcomes consistent with the literature on peer recovery support services, which shows that social support (alongside instrumental or practical support) is key in supporting recovery (Corrigan, 2024; Eddie et al., 2019; Kang & Kang, 2022). These findings are in line with the NDIS Review (2023) which advocates for a new approach to psychosocial disability in the NDIS based on personal recovery and optimising independence.

While psychosocial recovery coaching has been found to achieve positive outcomes, it is imperative that any future iterations of this support also work to address barriers to successful outcomes. Our findings on barriers to achieving outcomes align with previous research that has explored the impact of social factors such as limited social and financial resources on recovery (Tew et al., 2011), and barriers for people with psychosocial disability in accessing the NDIS – including social inequities; stigma, trauma and previous negative experiences; challenges accessing and understanding the Scheme; and mental health-related barriers (Mellifont et al., 2023; Roberts et al., 2024; Wilson et al., 2022).

Mental-health related barriers were identified in this study, despite finding that psychosocial recovery coaching overall has had a positive impact on people’s mental health, self-reliance and resilience. There were, however, also high levels of ‘no change’ demonstrating the need to address mental health-related barriers to ensure greater support for people’s mental and emotional health. Gaps in mental health services and the disconnect between mental health services and the NDIS prevent people from being as well as they can be and maximizing life outcomes (Devine et al., 2022). To increase the chances of recovery, sufficient and timely clinical and community services must be provided alongside disability supports such as psychosocial recovery coaching. The need to address a lack of clinicians, long waitlists and a shortage of community mental health services has been recognized (Commonwealth of Australia, 2023). Additional social barriers have been identified in a variety of research (van Weeghel et al., 2019; Wilson et al., 2022) and the need to continue to work to address these attitudinal and environmental barriers is incumbent upon the NDIS, government, providers and other social institutions to ensure the full and effective participation in society of people with psychosocial disability on an equal basis with others. Failure to do so can only limit the effectiveness of psychosocial recovery coaching.

Our findings highlight the relatively high proportion of people with psychosocial disability reporting inadequate services or service issues (36%) and issues with the NDIS (29%) as barriers to outcome achievement. These reflect findings from the NDIS Review that more can be done to support people with psychosocial disability in reaching self-determined life goals; and in accessing appropriate mental healthcare or other needed services (Commonwealth of Australia, 2023). NDIS participants have previously indicated a lack of choice and flexibility in accessing required services and uncertainty in what their funding entitled them to ask of such services (Wilson, et al., 2022). There are currently too few providers with psychosocial competencies and the NDIS is not stewarding the market to deliver a recovery-focused approach (Commonwealth of Australia, 2023).

One of the poorest outcome areas evidenced in the study was in the domain of employment with a high proportion indicating ‘no change’ (86%). This reflects the evidence that people with psychosocial disability experience some of the lowest labour force participation rates and highest unemployment rates compared to other people with disability and the general population (Devine et al., 2019). This despite the NDIS goal of supporting economic participation, with many people with psychosocial disability dissatisfied with the lack of funding provided to support their employment goals (Wilson et al., 2022). While caution needs to be applied to people indicating ‘no change’ given that there is not the data here to determine if people expected change in the context of psychosocial recovery coaching provision, it does highlight the need for further attention given the NDIS Review recommendations for appropriate supports for employment (Commonwealth of Australia, 2023) and the role that psychosocial recovery coaches could play in supporting people’s employment goals.

The specific types and roles of future NDIS psychosocial support provision are yet to be confirmed following the release of the NDIS Review Final Report, though a focus on personal recovery and strengthening the interface between mental health systems and the NDIS is prominent (Commonwealth of Australia, 2023). However, the NDIS Review has a recommendation for access to navigators (including specialist psychosocial recovery navigators) who have expertise in psychosocial supports and are trauma-informed. Navigators should work with participants to understand their life journey and ‘to connect with mental health services, education and employment’ (Commonwealth of Australia, 2023, p. 133).

This study’s findings highlight that such a role would require specialized psychosocial disability knowledge and an ability to build a trusted, consistent and positive relationship and to value and respect the people they work with. Peer relationships have been valuable in utilizing personal experience and ways of addressing challenges and coping with difficulties. Such a role also requires a willingness to listen to the people they support, to empower them to express their preferences and to engage in dialogue and feedback. These qualities of the psychosocial recovery coach shown to be valued by NDIS participants – and recognized in other recovery coaching literature – highlight the qualities and requirements for both the NDIS and service providers in ensuring positive support for people with psychosocial disability.

Our study on psychosocial recovery coaching provides evidence of the kinds of positive outcomes NDIS participants can achieve through connecting with a consistent contact who draws on lived and/or learned expertise in mental health to assist with support navigation and working towards personal recovery. These findings align with previous research on the benefits of peer recovery coaches (Eddie et al., 2019; Kang & Kang, 2022), highlighting the value of ensuring this form of support continues to be available for those with psychosocial disability, whether through the NDIS or other relevant schemes and programs. The NDIS and support services need to ensure that psychosocial recovery coaches continue to be available to support people with psychosocial disability and to ensure that the relevant qualities required are supported within the service context. Equally, attitudinal and environmental barriers need to be continually addressed to ensure the effectiveness of psychosocial recovery coaching to ensure ongoing positive outcomes.

## Limitations of the Study

The data in this report comes from a small sample of clients from a single psychosocial recovery coaching service within the context of the NDIS in Australia. In particular, the findings are skewed towards a Victorian cohort (55% of survey respondents and 90% of interviewees). As a result, some caution must be exercised when considering these results and the extent to which they reflect the outcomes of all psychosocial recovery coaching clients, as well as the applicability of findings outside of this specific cultural and service context. With both positive and negative outcomes, consideration must be given to the complexity of contextual factors impacting people’s lives and the experiences people reported in this data. It is not possible to account for all variables that may influence outcomes. Further, in our analysis it was evident that outcomes and concept definitions within each framework (CSOT, CHIME-D) defy precise and rigid definitions, making unambiguous alignment open to interpretation. Equally, the data speaks across multiple definitions and is contingent on interpretative analysis. While acknowledging this, researchers have made efforts to ensure reliability of the data and to limit potential biases, as previously discussed. However, given psychosocial recovery coaching’s recency and the limited evidence currently available, this research provides insights into clients’ experience and outcomes of psychosocial recovery coaching, and the learning that could be carried forward into future iterations of NDIS psychosocial supports.

## Conclusion

Our study shows that overall, participant experiences and outcomes of psychosocial recovery coaching align strongly with the CHIME-D recovery framework and demonstrate outcomes consistent with the NDIA stipulated goals and responsibilities of psychosocial recovery coaching service delivery.

This paper contributes new knowledge on the outcomes of psychosocial recovery coaching, while situating this service type in relation to existing bodies of work on peer-delivered services and peer recovery support services for people with substance use disorder and in mental health recovery. As previous research has advocated, our study focuses on personal, rather than clinical recovery, and draws on clients’ self-reported experience to better understand what outcomes change through psychosocial recovery coaching, as well as what barriers impede change. We find that psychosocial recovery coaching can deliver several positive outcomes in alignment with the CHIME-D recovery framework (van Weeghel et al., 2019).

The findings presented in this paper provide insights into the kinds of outcomes participants identify and value as part of a recovery-oriented NDIS service. Our study and other research (e.g. Corrigan et al., 2022) suggests that future iterations of NDIS psychosocial supports should align with these kinds of personal recovery outcomes as identified by people with psychosocial disability.

## Data Availability

Data is not publicly available as consent has not been provided for data to be shared beyond the named researchers on the approved ethics application.
